# Establishment of a novel tick-*Babesia* experimental infection model

**DOI:** 10.1038/srep37039

**Published:** 2016-11-14

**Authors:** Hiroki Maeda, Takeshi Hatta, M Abdul Alim, Daigo Tsubokawa, Fusako Mikami, Makoto Matsubayashi, Takeharu Miyoshi, Rika Umemiya-Shirafuji, Shin-ichiro Kawazu, Ikuo Igarashi, Masami Mochizuki, Naotoshi Tsuji, Tetsuya Tanaka

**Affiliations:** 1 Laboratory of Infectious Diseases, Joint Faculty of Veterinary Medicine, Kagoshima University, Korimoto, Kagoshima 890-0065, Japan; 2 Department of Pathological and Preventive Veterinary Science, The United Graduate School of Veterinary Science, Yamaguchi University, Yoshida, Yamaguchi 753-8515, Japan; 3 Department of Parasitology, Kitasato University School of Medicine, Kitasato, Minami, Sagamihara, Kanagawa 252-0374, Japan; 4 Department of Parasitology, Faculty of Veterinary Science, Bangladesh Agricultural University, Mymensingh-2202, Bangladesh; 5 National Institute of Animal Health, Kannondai, Tsukuba, Ibaraki 305-0856, Japan; 6 Laboratory of International Prevention of Epidemics, Graduate School of Life and Environmental Sciences, Osaka Prefecture University, Gakuen-cho, Nakaku, Sakai, Osaka 599-8531, Japan; 7 National Research Center for Protozoan Diseases, Obihiro University of Agriculture and Veterinary Medicine, Inada, Obihiro, Hokkaido 080-8555, Japan

## Abstract

Ticks are potent vectors of many deadly human and animal pathogens. Tick-borne babesiosis is a well-recognized malaria-like disease that occurs worldwide and recently has attracted increased attention as an emerging zoonosis. Although the proliferation of *Babesia* organisms is essential in the vectors, their detailed lifecycle with time information for migration in ticks remains unknown. A novel study model for the elucidation of the migration speed of *Babesia* parasites in their vector tick, *Haemaphysalis longicornis*, has been developed using an artificial feeding system with quantitative PCR method. The detectable DNA of *Babesia* parasites gradually disappeared in the tick midgut at 1 day post engorgement (DPE), and in contrary increased in other organs. The results indicated that the *Babesia* parasite passed the *H. longicornis* midgut within 24 hours post engorgement, migrated to the hemolymph, and then proliferated in the organs except the midgut. This time point may be an important curfew for *Babesia* parasites to migrate in the tick lumen. We also visualized the *Babesia* parasites in the experimentally infected ticks and in their eggs using IFAT for detecting their cytoskeletal structure, which suggested the successful tick infection and transovarial transmission of the parasite. This model will shed light on the further understanding of tick-*Babesia* interactions.

Ticks are notorious hematophagous ectoparasites of almost terrestrial vertebrates and well known as a unique vector of various deadly diseases, such as Lyme borreliosis, tularemia, anaplasmosis, babesiosis, theileriosis, tick-borne encephalitis, and severe fever with thrombocytopenia syndrome (SFTS)^1^,^2^. About 900 tick species, including approximately 700 ixodids and 200 argasids, are distributed throughout the world^3^. Recent analysis of the tick microbiome indicates that ticks harbor a wide variety of microorganisms^4^. Pathogens, including bacteria, protozoa, and viruses, are taken up with the blood meal and exposed to a potentially hostile environment in the tick’s midgut before they invade the gut cells. It is assumed that the tick-pathogen interaction in relation to the adaptation and proliferation of the pathogens in ticks and their successful transmission to the vertebrate hosts is maintained by molecular mechanisms^5^,^6^. It has been shown that the bioactive molecules such as longicin and longipain from *H. longicornis* critically regulates the transmission of *Babesia* parasites in the tick^7^,^8^.

Babesiosis is caused by intraerythrocytic apicomplexan parasites which belong to the genus *Babesia* and is mainly transmitted by tick vectors to a variety of vertebrate hosts, including wild and domestic animals and also humans^9^,^10^. *Babesia* species undergo a complex developmental cycle in the vertebrate host and tick, somewhat analogous to that of malaria parasites and their mosquito vector. With the worldwide distribution of ixodid ticks, babesiosis is the second most common blood-borne disease of mammals. The major tick vectors of *Babesia* globally are the *Rhipicephalus* and *Haemaphysalis* species^11^. The ixodid tick *H. longicornis*, one of the most important tick species in Asia and Australia, is a natural vector of the protozoa that causes babesiosis in humans and domestic animals^12^. *B. ovata* is a benign intraerythrocytic protozoan parasite of cattle which is vertically (transovarially) transmitted by *H. longicornis*. It has been experimentally proved that transmission of *B. ovata* to cattle takes place by only the larval stage of *H. longicornis*^13^ and it is considered that the larval stage of *H. longicornis* is the important stage for the transmission of babesiosis caused by *B. ovata*. Also, other cattle *Babesia* with high pathogenicity including *B. bigemina* and *B. bovis*, were transmitted transovarially^14^,^15^,^16^. *B. ovata* causes no severe clinical symptoms^11^ and it is considered to be a model organism for investigating the tick stage of *Babesia*. For the advancement of the understanding of the relationship between the vector and vector-borne pathogens, laboratory experimental models as with mosquito and malaria parasite^17^,^18^ might be produced. Several studies on the lifecycle of *Babesia* parasites in ticks have been conducted; however, unlike malaria parasites^19^, our knowledge about the time information for the development of *Babesia* parasites in the vector is still limited^20^,^21^. Moreover, when using experimental animals such as cattle, tick-*Babesia* research is expensive and requires great efforts including the clinical management of animals and rearing ticks and the maintenance of *Babesia* parasites in the laboratory.

Tick artificial feeding (membrane feeding or tube feeding) is a widely used technique that is considered as an effective tool for producing ticks infected with parasites^22^,^23^,^24^. This technique mimics the natural process of feeding and reduces the host factors. To date, many studies on the tick-pathogen interaction based on the artificial feeding system have been conducted^25^,^26^,^27^. However, for monitoring the transmission speed of the parasites in the tick vector by these systems, it costs us lot efforts to obtain the engorged ticks because of their long artificial-feeding period. Generally, female ticks stop their blood feeding partially and wait to mate with male ticks. After mating, the female ticks resume feeding toward engorgement and laying eggs^28^. Due to their unique feeding process, it is hard to synchronize their engorgement. Here, we report a novel tick-*Babesia* experimental infection model with *H. longicornis* and *B. ovata* using a semi-artificial feeding system^29^. In this report, we developed the quantification model for transovarially transmitted *Babesia* parasite in the vector ticks. This system is quite simple and we could obtain synchronous engorged tick experimentally infected with *Babesia* pathogens within 12-24 hours of artificial feeding period. Through this model it was possible to monitor quantitatively the transovarial transmission of *Babesia* parasites. This model will give insight toward the development of new control strategies of babesiosis.

## Results

### B. ovata migration in ticks

**Figure 1:**
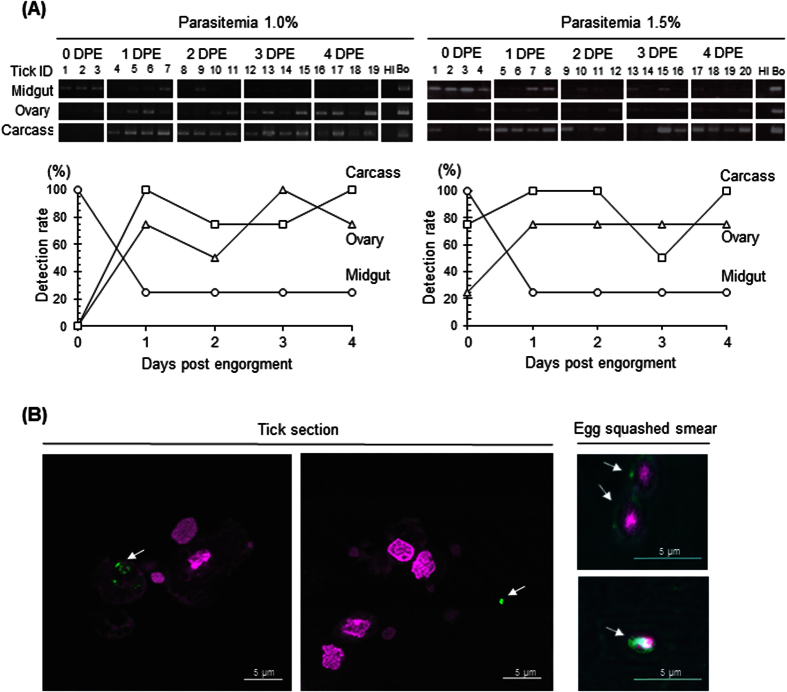
(**A**) Results of two experiments for the detection of *B. ovata* in tick organs. The top figure shows the conventional PCR. The lower line graph indicates the percentage of countable samples from qPCR. The same concentration of DNA was used for each sample. Tick and *B. ovata* DNA was prepared for negative and positive controls. DPE, days post engorgement; Hl, *H. longicornis*; Bo, *B. ovata*; 1–20, Tick ID No. (**B**) IFAT in the tick samples. The two left panels show *B. ovata* in the tick sections. Arrows indicate the *B. ovata*. The two right panels show *B. ovata* parasites (arrows) in the egg squashed smears. Bar: 5 μm.

The conventional PCR results in Fig. 1A show the existence of *B. ovata β-tubulin* DNA in the tick organs collected at the indicated days after their engorgement. The positive bands of *B. ovata* in the midgut samples seemed to gradually disappear after 1 day post engorgement (DPE). On the contrary, those in the ovary and carcass were likely to increase after 1 DPE. Two independent trials showed the same tendency. To quantify the parasite burden in the samples, qPCR was also conducted. Initially, we verified the sensitivity of the qPCR and confirmed the typical amplification curve, melting curve with no primerdimerization, and correlative standard curve using the artificially prepared meta-genomic DNA samples, including the plasmid DNA-carrying *B. ovata β-tubulin* fragment and tick genomic DNA (Supplementary Fig. 1). The developed qPCR system was able to detect and quantify the parasite DNA in some samples; however, the values of the parasite burden in the qPCR-positive samples were quite low and did not show any correlation between their values and body weight (Supplementary Table 2). However, the detection rate calculated from the results of qPCR showed the same tendency as the results of conventional PCR. The detection rate in the midgut at 0 DPE was 100%, that decreased to 25% at 1 DPE and onward. In contrast, the detection rate in the ovary and carcass samples reached to 75–100% at 1 DPE and onward (Fig. 1A). These results imply that *B. ovata* might pass through the midgut epithelium within 24 hours after tick engorgement.

### Localization of Babesia parasites in tick organs

To detect the parasite in the ticks specifically, *B. ovata* P29 (BoP29; Accession No. LC110193; Supplementary Fig. 2), homologous to a cytoskeleton protein and conserved among the other apicomplexan protozoa; *Toxoplasma gondii*^30^ and *B. gibsoni*^31^ was selected as marker for the IFAT. P29 protein is one of the important cytoskeleton proteins in the apicomplexan parasites and, thus, is considered to be their constitutive protein. Six histidine-tagged recombinant BoP29 were expressed using *E. coli* for the preparation of specific antisera against BoP29 (Supplementary Fig. 3A). As shown in the immunoblotting result (Supplementary Fig. 3B), the anti-BoP29 mouse serum detected a clear 29-kDa single band in the lysate of *B. ovata*-infected RBCs. Furthermore, anti-BoP29 specifically reacted with a fiber-like structure of the *B. ovata* merozoite in the parasitized RBCs on the blood smear (Supplementary Fig. 4). In the tick sections, dot-like reactions identical to reactions to the body parts of *B. ovata* parasites were detected in the tick cells or hemocoel (Fig. 1B). This suggests that because the sections were cut, positive fluorescence indicating BoP29 was detected in the cells and in the hemocoel apart from other organs. We were limited in that we could not identify the origin of the cells; furthermore, detection of the complete form of *B. ovata* parasites was difficult. As shown in the previous report^32^, spherical cells with a positive signal were also observed in the egg squashed smear (Fig. 1B), which strongly suggests that *B. ovata* infected *H. longicornis* ticks and transovarially migrated to their eggs.

## Discussion

**Figure 2:**
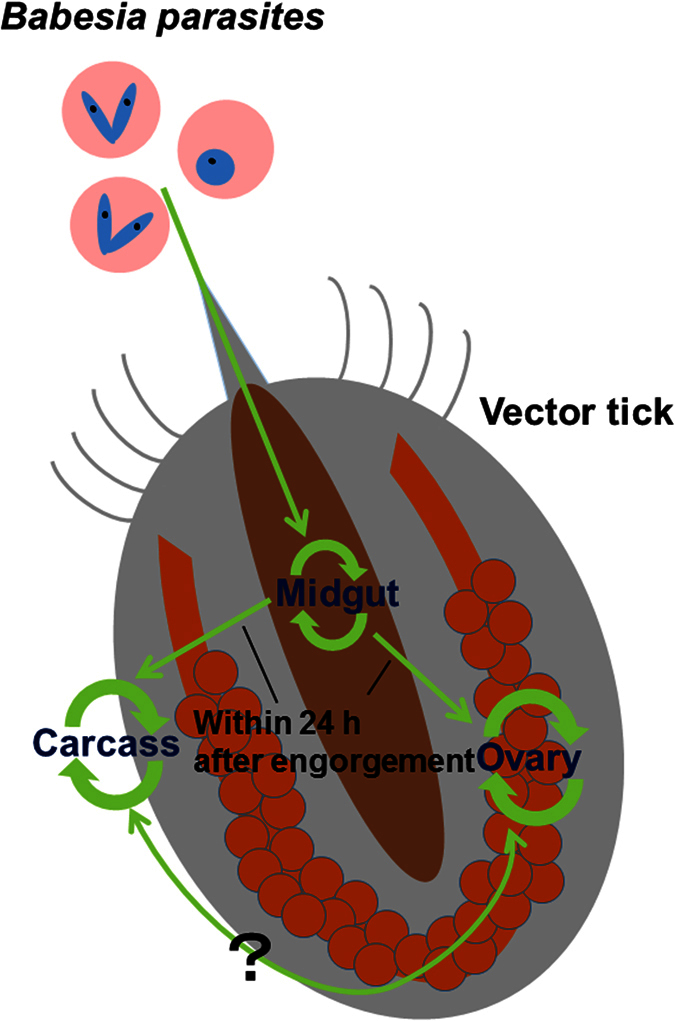
Conclusive figure. Green arrows show the migration kinetics of *B. ovata. B. ovata* might pass through the midgut epithelium within 24 h after tick engorgement, and then proliferation will occur in organs except the midgut.

The three-host tick, *H. longicornis*, is very useful and suitable for studying the transovarial transmission of *Babesia* parasites because this tick has a unique thelytokous parthenogenetic characteristic. This tick does not need to mate for its engorgement and reproduction^28^. *H. longicornis* is easy to maintain and is widely used as a model tick to study pathophysiology in tick infestation^33^. To date, a number of bioactive molecules have been found in *H. longicornis*, and those products might be available for not only tick control but also for novel drug discovery in the veterinary and medical fields^7^,^8^,^34^,^35^,^36^. The experimental infection of ticks with pathogens is considered to be an attractive tool for studying tick-pathogen interaction^5^,^6^. In the present study, a novel tick-*Babesia* experimental model was established and validated. Interestingly, a reverse phenomenon for the detection rate of *B. ovata* of the midgut sample as compared with the ovary/carcass samples was observed at 1 DPE. In some studies, a positive correlation between the blood parasite levels of infected animals and the kinete level of *Babesia* in the hemolymph of the dropped tick has been shown^11^,^15^,^16^. We applied *B. ovata*-infected RBCs with parasitemia of 1–2%, which was assumed to be approximately 100 times higher than that of the natural condition (less than 0.01%)^11^. Nevertheless, *B. ovata* DNA in some samples was not detected by qPCR. This might be caused by significant *in vitro* passage of *B. ovata*, resulting in phenotypic changes regarding the ability of parasites to infect the vector tick, as described in previous articles^37^,^38^,^39^. In accordance with our evidence, we hypothesized that most *Babesia* parasites might pass through the midgut barrier within 24 hours after tick engorgement, and the direction of their migration at the midgut epithelium might be one way (Fig. 2). Gough *et al*.^40^ showed a time-course model of a stage transition of *B. bigemina* in the midgut lumen of its vector tick, *Rhipicephalus (Boophilus*) *microplus*^40^. They cultured merozoites of *B. bigemina in vitro* with the midgut extract from the engorged female ticks and showed that its development from the merozoite to the zygote finished within 7.5 hours post cultivation. Bock *et al*.^41^ reviewed that the zygote selectively infects the digestive cells and vitellogenic cells of the tick midgut and that its multiplication probably occurs in those cells to develop kinetes that move into the tick hemolymph^41^. These reports partly support our hypothesis that 24 hours after engorgement is considered as an important window for the migration of *B. ovata* in the tick lumen. In addition, we detected the *B. ovata* DNA from tick ovaries and *Babesia* parasites in the *B. ovata*-infected tick eggs, which supported the transovarial transmission of this parasites (Fig. 1). Higuchi *et al*.^42^ first detected the *B. ovata* from ovary and eggs of *H. longicornis* dropped from the *Babesia*-infected cattle^42^. They detected kinetes for the first time at 6 DPE. However, as shown in the Fig. 1A, qPCR-based assay proved that after 1 DPE, tick ovary gradually become pathogenic. It is considered that this system is more sensitive than the microscopic method. We also observed the round-formed bodies of *Babesia* parasites in the tick eggs as previously reported^32^,^42^. The morphological changes of *Babesia* parasites were also detected in the midgut of nymphal stages of *H. longicornis* fed on infected cattle by Higuchi *et al*.^43^. The similar development of *Babesia* parasites was observed in the midgut of artificially-engorged adult ticks in this study (data not shown). The successful development and migration of *B. ovata* in ticks indicated that the *in vitro* cultured pathogens conserved the transmission ability.

A novel system for evaluating the interaction between *B. ovata* and its vector tick, *H. longicornis*, has been developed. This method is considered to be quite simple and cost effective and could be used to monitor and quantify the infection level of *B. ovata* in tick organs with high sensitivity. Our experimental model would be a powerful tool for clarifying the kinetics of the tick stage of *Babesia* parasites. With the rapid progress of genome-editing strategies, transgenic organisms are currently available as attractive tools to aid in molecular and cellular studies of *Babesia* and *H. longicornis*^34^,^44^. Precise study of tick-*Babesia* molecular interactions using our developed model will also give us concrete knowledge to develop novel strategies for controlling babesiosis.

## Methods

### Ticks and mice

The parthenogenetic Okayama strain of *H. longicornis*^12^ was maintained by blood feeding on 6-month-old BALB/c mice (Japan SLC, Shizuoka, Japan) at the Department of Parasitology, Kitasato University School of Medicine. BALB/c mice were cared for in accordance with the guidelines approved by the Animal Care and Use Committee of Kitasato University (Approval No. 2015171). They were maintained in regulated conditions throughout the experiments.

### *In vitro* culture of B. ovata

The *in vitro* microaerophilus stationary phase culture system of *B. ovata* (Miyake strain) established by Igarashi *et al*.^45^ was slightly modified to reduce the unknown factors in the bovine serum. GIT (Nihon Pharmaceutical Co., Ltd., Tokyo, Japan) instead of bovine serum and M199 medium (Sigma-Aldrich, St. Louis, MO, USA) were used as the mixture with a ratio of 2:3. Fresh bovine blood was purchased from Nippon Bio-Test Laboratories Inc. (Tokyo, Japan) to prepare the red blood cells (RBCs)^35^. The culture was maintained at 37 °C with 5% oxygen and 5% carbon dioxide. Giemsa-stained blood smears were examined daily to determine the parasitemia, which was calculated as the percentage of parasitized RBCs for 1,000 total RBCs counted. *B. ovata*-infected RBCs of approximately 1–2% parasitemia were used to feed ticks artificially.

### DNA extraction from artificially engorged tick organs

To prepare the mouse skin membrane, female adult ticks were allowed to feed on the shaven back of BALB/c mice. 8 to 10 ticks were attached on each mice. After 4 to 5 days (at the beginning of the expansion period), a rectangular section of the mouse skin with the ticks attached was carefully removed from the mouse’s body immediately after euthanasia, and set on the artificial feeding units^29^. Inside of the membrane was washed with sterilized phosphate-buffered saline (PBS) supplemented with 100 units/ml penicillin and 100 μg/ml streptomycin (Life Technologies Corporation, Carlsbad, CA). The ticks were fed on the RBC solution, composed of the fresh media and *B. ovata*-infected RBCs at a ratio of 7:3, using the artificial feeding units. To serve and maintain the fresh parasites in the system, the RBC solution that included *B. ovata* was changed every 12 hours during tick feeding. Engorged and dropped ticks (19 from the experiment with approximately 1% parasitemia of *B. ovata* or 20 from the experiment with approximately 1.5% parasitemia) were obtained, and 3–4 of them were dissected daily from 0 to 4 days post engorgement (DPE) to pick off the midgut, ovary, and carcass that included other organs. These organs were rinsed with PBS and immediately put into the DNA extraction buffer to extract the DNA as described previously^35^. The concentration of purified DNA was determined by NanoDrop 2000 (Thermo Scientific, Waltham, MA, USA) and then diluted at 50 ng/μl and stored at −30 °C until use.

### PCR detection of B. ovata DNA in the tick

A specific primer set for *B. ovata β-tubulin* gene^46^ (Supplementary Table 1) was used in this study. For conventional PCR, KOD-Plus-Neo (Toyobo, Osaka, Japan) was used. The cycling conditions were as follows: initial denaturation at 95 °C for 2 min, followed by 40 cycles of denaturation at 98 °C for 10 sec, annealing at 60 °C for 30 sec, extension at 68 °C for 15 sec, and final extension at 68 °C for 7 min. Quantitative PCR (qPCR) was also performed with LightCycler 1.5 (Roche, Basel, Switzerland) using KOD SYBR qPCR Mix (Toyobo). For the standard templates, the *B. ovata* β-tubulin fragment was cloned into the pTA2 vector (Bo β-tub/pTA2) using a Mighty TA-cloning Kit (TaKaRa, Shiga, Japan). The cloned sequence was confirmed using the DNA-sequencing service of FASMAC Co., Ltd. (Kanagawa, Japan). To linearize the super-coiled plasmids, the plasmid DNA was treated with a *Bam*HI restriction enzyme (Toyobo). The copy number of 1 μg of the plasmid DNA was calculated as the result of 9.1 × 10^11^ divided by the size of plasmid DNA (kb). The standard template was a series diluted 10-fold with 50 ng/μl of tick DNA. The PCR cycling steps were as follows: initial denaturation at 98 °C for 2 min, followed by 40 cycles of denaturation at 98 °C for 10 sec, annealing at 60 °C for 10 sec, and extension at 68 °C for 30 sec. For the tick internal control, HlITS2 was used, as reported previously^32^ (Supplementary Table 1). The parasite burden was quantified as the ratio of the amplicon of the *B. ovata* β-tubulin fragment to the tick HlITS2 fragment for each sample. All test samples and plasmid standards were assayed in duplicate. For each PCR template, 50 ng of DNA/reaction was used. The detection rate was calculated as the percentage of the number of quantified samples in each group.

### Visualization of B. ovata in the tick

Indirect immunofluorescent antibody test (IFAT) using tick section was performed as described previously^34^. Briefly, ticks were fixed overnight in a 4% paraformaldehyde phosphate buffer solution (pH 7.4) that included 0.1% glutaraldehyde and was embedded in paraffin. Cut sections were fixed on glass slides and deparaffinized in xylene. The sections were rehydrated with a graded series of alcohol and PBS, followed by trypsin treatment. They were then blocked using Blocking One Histo (Nacalai Tesque, Kyoto, Japan). They were then incubated for 1 hour at 37 °C with mouse anti-BoP29 serum (1:100) diluted by Can Get Signal Immunostain Immunoreaction Enhancer Solution (Toyobo). Sections treated with pre-immune mouse sera (1:100) were used as a control. After washing, sections were reacted with Alexa Fluor 488 goat anti-mouse IgG as a secondary antibody (1:1000) and mounted with VECTASHIELD Mounting Medium with DAPI (Vector Laboratories, Burlingame, CA, USA). The slides were examined under a confocal laser scanning microscope (LSM 710, Carl Zeiss, Oberkochen, Germany) with LSM Software ZEN 2012 (Carl Zeiss). For the IFAT with egg squashed smear, randomly selected eggs of 10 days post oviposition were used and treated as described previously^32^.

## Additional Information

**How to cite this article**: Maeda, H. *et al*. Establishment of a novel tick-*Babesia* experimental infection model. *Sci. Rep.* **6**, 37039; doi: 10.1038/srep37039 (2016).

**Publisher's note:** Springer Nature remains neutral with regard to jurisdictional claims in published maps and institutional affiliations.

## Supplementary Material

Supplementary Information
