# Supplementary material for: Establishment of a novel tick-Babesia experimental infection model
Source: Sci Rep. 2016 Nov 14;6:37039. doi: 10.1038/srep37039 (PMC5107930; doi:10.1038/srep37039)
Supplement: Supplementary Information [file srep37039-s1.pdf]

Establishment of a novel tick-Babesia experimental infection model

Hiroki Maeda[1,2], Takeshi Hatta[3,*], M Abdul Alim[4,5], Daigo Tsubokawa[3], Fusako Mikami[3], Makoto Matsubayashi[6], Takeharu Miyoshi[5], Rika Umemiya-Shirafuji[7], Shin-ichiro Kawazu[7], Ikuo Igarashi[7], Masami Mochizuki[1,2], Naotoshi Tsuji[3], Tetsuya Tanaka[1,2,*]

[1]Laboratory of Infectious Diseases, Joint Faculty of Veterinary Medicine, Kagoshima University, Korimoto, Kagoshima 890-0065, Japan
[2]Department of Pathological and Preventive Veterinary Science, The United Graduate School of Veterinary Science, Yamaguchi University, Yoshida, Yamaguchi 753-8515, Japan
[3]Department of Parasitology, Kitasato University School of Medicine, Kitasato, Minami, Sagamihara, Kanagawa 252-0374, Japan
[4]Department of Parasitology, Faculty of Veterinary Science, Bangladesh Agricultural University, Mymensingh-2202, Bangladesh
[5]National Institute of Animal Health, Kannondai, Tsukuba, Ibaraki 305-0856, Japan
[6]Laboratory of International Prevention of Epidemics, Graduate School of Life and Environmental Sciences, Osaka Prefecture University, Gakuen-cho, Nakaku, Sakai, Osaka 599-8531, Japan
[7]National Research Center for Protozoan Diseases, Obihiro University of Agriculture and Veterinary Medicine, Inada, Obihiro, Hokkaido 080-8555, Japan

*Correspondence and requests for materials should be addressed to
T.H. (email: htakeshi@med.kitasato-u.ac.jp) or T.T. (e-mail: tetsuya@ms.kagoshima-u.ac.jp)

Supplementary Methods

Recombinant protein expression of *B. ovata* P29

The predicted P29 homologous fragments were amplified from *B. ovata* cDNA using the result of RNA sequencing from the midgut of *B. ovata*-infected ticks at 3 DPE (unpublished data). After confirming the sequencing (Supplementary Fig. 2, FASMAC Co., Ltd.), the sequence was cloned into the pRSET B vector (Invitrogen, Carlsbad, CA, USA) using an In-Fusion® HD Cloning Kit (TaKaRa) with a specific primer set (Supplementary Table 1). For In-Fusion cloning, the pRSET B vector was digested with *Bam*HI and *Hin*dIII restriction enzymes (Toyobo) and purified with the MinElute Gel Extraction Kit (Qiagen, Hilden, Germany).

The recombinant plasmid was transformed in Rosetta™ (DE3) Competent Cells (Novagen, Darmstadt, Germany) and expressed by induction with 1 mM isopropyl-β-D(-)-thiogalactopyranoside (IPTG) at 37°C for 4 h. The expressed recombinant protein was purified using the ProBond Purification System (Invitrogen) from the insoluble fraction under denaturing conditions. The purified recombinant protein was dialyzed against the PBS solution for refolding. The purity of the recombinant proteins was checked by SDS-PAGE. The concentrations of recombinant proteins were determined by a Micro BCA™ Protein Assay Kit (Thermo Scientific) and stored at -30°C until use.

Production of an anti-serum against *B. ovata* P29

To prepare mouse anti-BoP29 sera, 100 μg of recombinant BoP29 completely mixed with TiterMax Gold Adjuvant (Sigma-Aldrich) was *subcutaneous*ly injected into BALB/c mice (Japan SLC). After 2 weeks, these mice were injected again to boost the generation of antibodies. Whole blood was collected from the mice 2 weeks after the second immunization to obtain the specific antisera for BoP29.

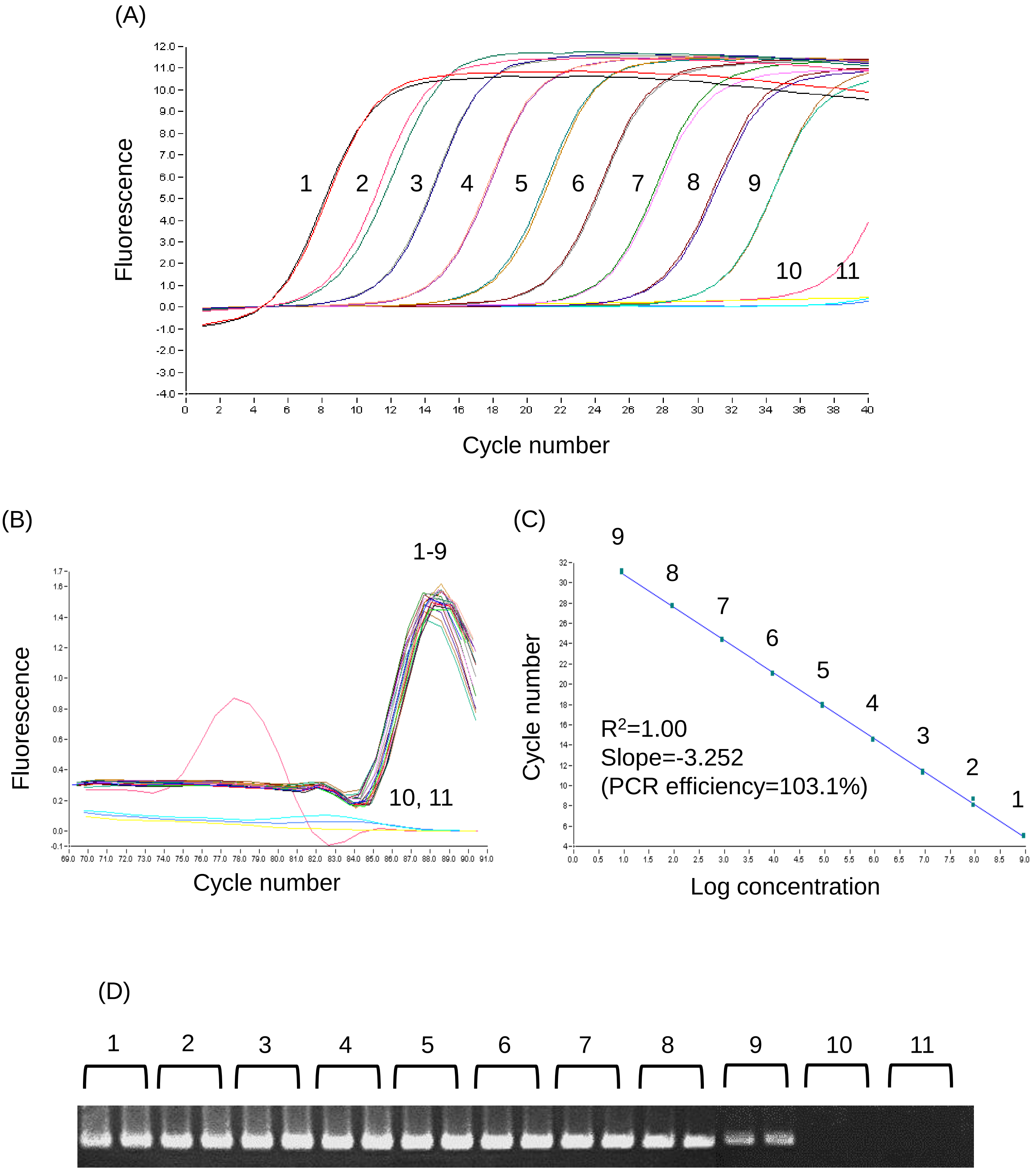

Supplementary Fig. 1. Sensitivity of qPCR. To confirm the detection limit of qPCR, a 10-fold serial dilution of Bo β-tub/pTA2 was used for a template. Tick DNA and water were also used for a negative control. (A) Amplification curve (B) Melting curve (C) Standard curve (D) Gel electrophoresis of the PCR products. 1–9, 10-fold serial dilutions of Bo β-tub/pTA2 plasmids mixed with tick genomic DNA; 10, tick DNA; 11, water sample.

```
  1 ATGCAGTGCTGTTCTAGGGACACTCGTCTAATGGACGAGGAGATCGACACCCAAGACGTA
     M  Q  C  C  S  R  D  T  R  L  M  D  E  E  I  D  T  Q  D  V  20

 61 CAAGTGAGGACAATCGGCACCGTGGCCGACCGTTCCAGGGCACAAGAAATTGGCCAAAAC
     Q  V  R  T  I  G  T  V  A  D  R  S  R  A  Q  E  I  G  Q  N  40

121 GTCGAGAGGCAATGGGTCGCCGTTACCACCTACCAGCCCGTTGATACCATTACGAAGACG
     V  E  R  Q  W  V  A  V  T  T  Y  Q  P  V  D  T  I  T  K  T  60

181 GTGGAAATTCCAGTTGTCAAGACCGTTGAACGCGTTGTCCCCAAGCCTGTCATCCAGGAA
     V  E  I  P  V  V  K  T  V  E  R  V  V  P  K  P  V  I  Q  E  80

241 CGTGTAATTCAGGTGCCCCGCGAAGTTACCCAGGTTGTTGAAAAGGTTGTTGAGATCCCT
     R  V  I  Q  V  P  R  E  V  T  Q  V  V  E  K  V  V  E  I  P  100

301 GATGTAAAGTTCGTCGAGAAGATCATTGAGGTTCCACAGGTCCAATACCGCAACAAACTC
     D  V  K  F  V  E  K  I  I  E  V  P  Q  V  Q  Y  R  N  K  L  120

361 GTACCGAAGGTTGAGGTTGTTGAGAAGATCGTGGAAAAGCCGCAAATCATCGAGCAGTGG
     V  P  K  V  E  V  V  E  K  I  V  E  K  P  Q  I  I  E  Q  W  140

421 ACTGAGCGCAAGGTCGAGGTTCCCCAAATTAAGGAGGTTGTGCGCTACAAGGAAATTGAT
     T  E  R  K  V  E  V  P  Q  I  K  E  V  V  R  Y  K  E  I  D  160

481 GAGACAGAGGAGATCATCCGCTACTACCCTAAGGGACATGGCAACATTGACTGGGATAAG
     E  T  E  E  I  I  R  Y  Y  P  K  G  H  G  N  I  D  W  D  K  180

541 GAGTGCGAAAAGGCTCACATTATGATTCCCAGCGAGGTTACGGAATCCAAGGCCGCTAAC
     E  C  E  K  A  H  I  M  I  P  S  E  V  T  E  S  K  A  A  N  200

601 AAGCCCGAGGCAACTGCTTAA
     K  P  E  A  T  A  *  207
```

Supplementary Fig. 2. Cloned P29 ORF sequences from *B. ovata.* Cloned BoP29 ORF consists of 621 bp encoding 206 amino acids and shows high homology with *Babesia bigemina* P29 (99%), *Babesia bovis* P29 (93%), and *Babesia gibsoni* P29 (89%).

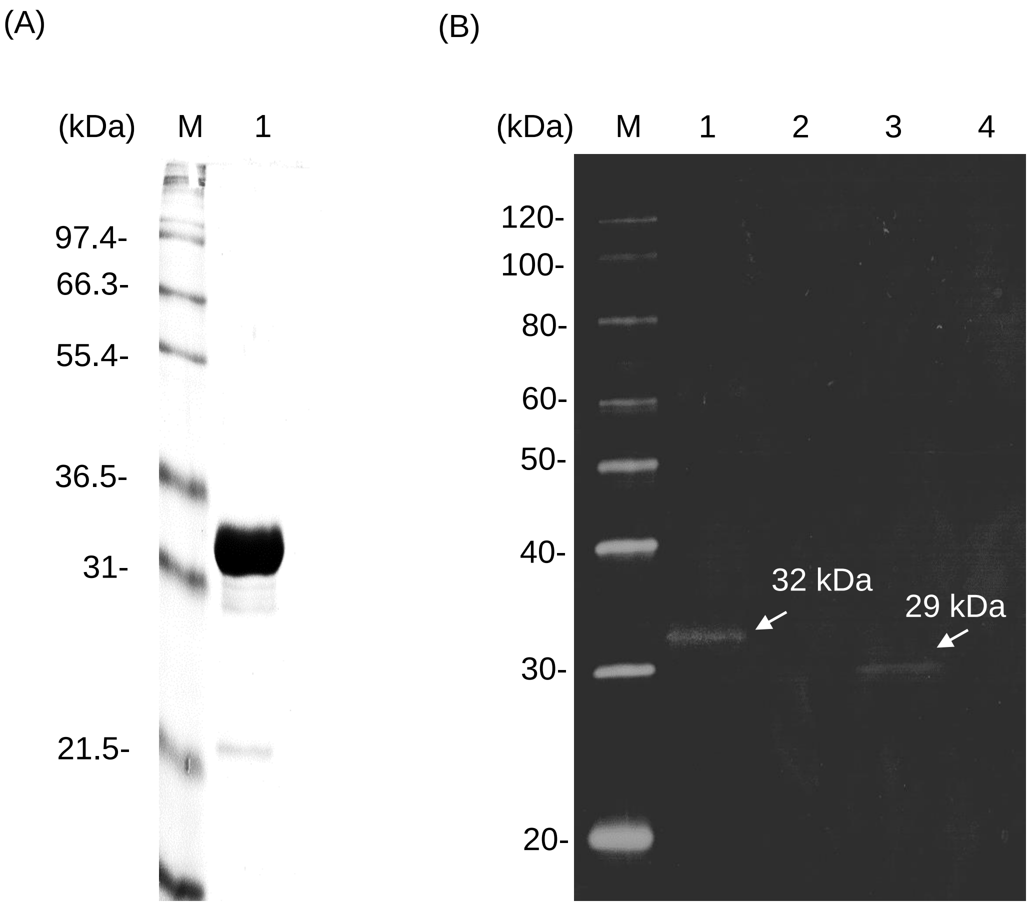

Supplementary Fig. 3. (A) Purified recombinant BoP29 loaded in 15% SDS-PAGE gel. (B) Sensitivity and specificity of anti-BoP29 mouse serum by western blotting. Lane 1: purified recombinant BoP29; Lane 2: *E. coli* lysate with expression vector; Lane 3: lysate of *B. ovata*-infected RBCs; Lane 4: lysate of normal RBCs.

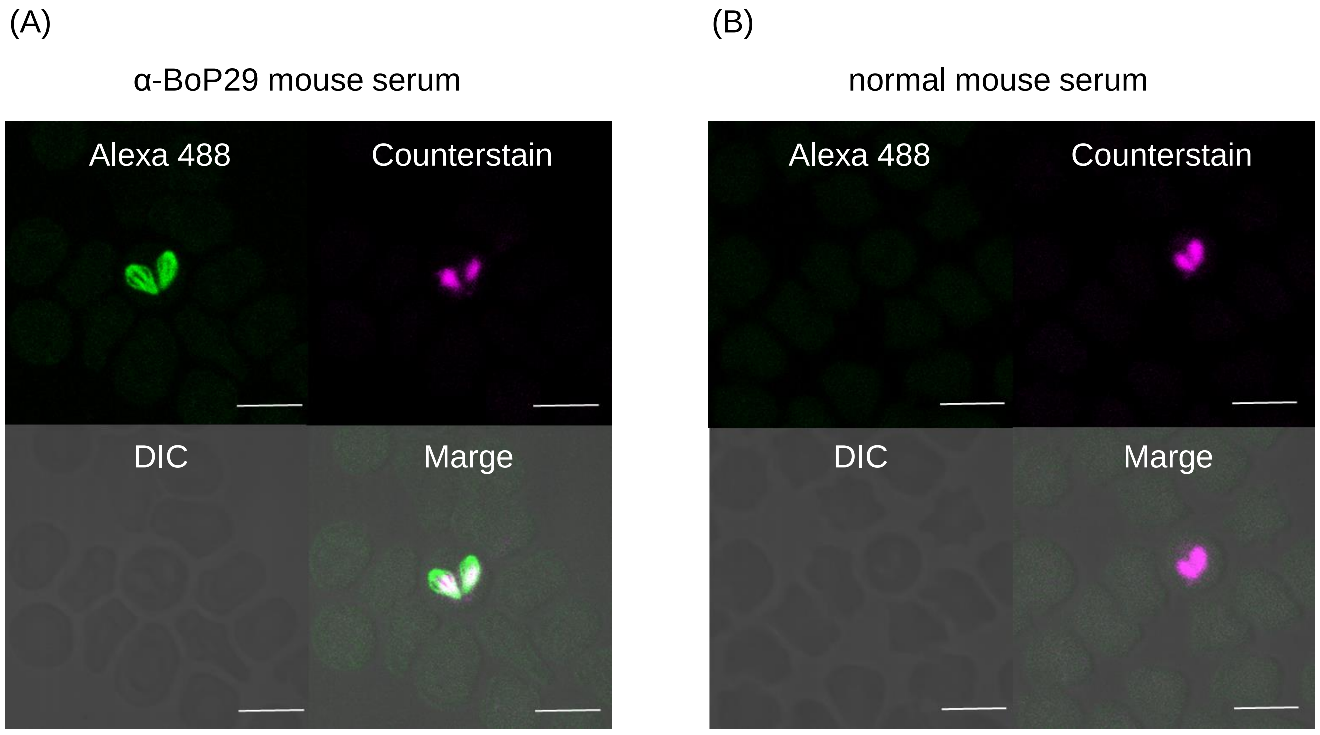

Supplementary Fig. 4. IFAT of *B. ovata*-infected RBCs (A) anti-BoP29 mouse serum (B) normal mouse serum. Bar: 5 μm

Supplementary Table 1 Gene-specific primers used in this paper

Primers	Sequence (5’→3’)
Bob-tub F[a)]	ACACTGTGCATCCTCACCGTCATAT
Bob-tub R[a)]	CTCGCGGATCTTGCTGATCAGCAGA
HlITS2 F[b)]	GCGTGTTGGGAAGTCTGAA
HlITS2 R[b)]	CGCGGTTTACGAGAGAAAG
BoP29 in-fusion F	ATGACGATAAGGATCCGATGCAGTGCTGTTCTAGGG
BoP29 in-fusion R	GCCAAGCTTCGAATTTTAAGCAGTTGCCTCGGG

[a)]Sivakumar et al, 2014; [b)]Hatta et al, 2012

Supplementary Table 2 Parasite burden of *B. ovata* in tick organs

DPE	Experiment 1					Experiment 2				
	Tick ID	BW (mg)	MG	OV	CA	Tick ID	BW (mg)	MG	OV	CA
0	#1	123.3	+	ND	ND	#1	105.1	+	ND	+
	#2	120.9	+	ND	ND	#2	92.8	+	+	ND
	#3	214.7	+	ND	ND	#3	235.9	++	ND	+
						#4	181.4	+	ND	+
1	#4	220.9	ND	ND	++	#5	147.1	ND	ND	+
	#5	241.4	ND	+	+	#6	151.4	ND	++	++
	#6	159.6	ND	++	+	#7	179.9	ND	+++	+
	#7	150.2	++	++	++	#8	202.8	+	++	+
2	#8	188.5	ND	ND	+	#9	154.1	ND	+	+
	#9	277.4	++	ND	+	#10	186.1	ND	ND	+
	#10	145.9	ND	+	+	#11	211.3	ND	++	+
	#11	183.6	ND	++	ND	#12	193.5	+	++	+
3	#12	165.2	ND	+	ND	#13	230.2	ND	ND	ND
	#13	139.4	++	+	++	#14	199.1	ND	++	ND
	#14	173.4	ND	+	+	#15	191.6	ND	++	++
	#15	184.6	ND	++	ND	#16	267.5	+	++	+
4	#16	265.9	ND	++	++	#17	274.3	ND	+	++
	#17	166.6	ND	+	+	#18	229.3	ND	+	++
	#18	160.9	ND	ND	+	#19	220.5	+	+	++
	#19	127.9	++	++	++	#20	253.1	ND	ND	+

The quantified copy number of the *B. ovata β-tubulin* gene was normalized by the tick *ITS2* gene. The quantified value was presented as +, 10^{-9}~10^{-6}; ++, 10^{-6}~10^{-3}; +++, 10^{-3}~10^{0}. ND, not detected
